# Morphological, cytological, and molecular evidences for natural hybridization between *Roegneria stricta* and *Roegneria turczaninovii* (Triticeae: Poaceae)

**DOI:** 10.1002/ece3.8517

**Published:** 2022-01-12

**Authors:** Chen Chen, Zilue Zheng, Dandan Wu, Lu Tan, Cairong Yang, Songqing Liu, Jiale Lu, Yiran Cheng, Lina Sha, Yi Wang, Houyang Kang, Xing Fan, Yonghong Zhou, Changbing Zhang, Haiqin Zhang

**Affiliations:** ^1^ Triticeae Research Institute Sichuan Agricultural University Chengdu China; ^2^ State Key Laboratory of Crop Gene Exploration and Utilization in Southwest China Sichuan Agricultural University Chengdu China; ^3^ College of Chemistry and Life Sciences Chengdu Normal University Chengdu China; ^4^ College of Grassland Science and Technology Sichuan Agricultural University Chengdu China; ^5^ Sichuan Academy of Grassland Science Chengdu China

**Keywords:** chromosome pairing, *DMC*1, FISH, GISH, natural hybrids, *Roegneria*, *rps*16

## Abstract

Some plants with low fertility are morphologically intermediate between *Roegneria stricta* and *Roegneria turczaninovii*, and were suspected to be natural hybrids between these species. In this study, karyotype analysis showed that natural hybrids and their putative parents were tetraploids (2n = 4x = 28). Meiotic pairing in natural hybrids is more irregular than its putative parents. Results of genomic in situ hybridization and fluorescence in situ hybridization indicate that natural hybrids contain the same genome as their putative parents. The nuclear gene DNA meiotic recombinase 1 (*DMC*1) and the chloroplast gene *rps*16 of natural hybrids and their putative parents were analyzed for evidence of hybridization. The results from molecular data supported by morphology and cytology demonstrated that the plants represent natural hybrids between *R. stricta* and *R*. *turczaninovii*. The study is important for understanding species evolution in the genus since it demonstrates for the first time the existence of populations of natural homoploid hybrids in *Roegneria*. The study also reports for the first time that the composition of the genomic formula of *R*. *turczaninovii* is **StY**, confirming that the current taxonomic status is correct.

## Graphical abstract

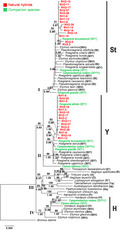

## 1 INTRODUCTION

Hybridization is the main driving force of plant evolution (Soltis & Soltis, 2009). It is estimated that about 25% of plant species are known to be involved in hybridization with other species (Mallet, 2005). These can provide source of genetic variation than on further evolution, through adaptation and selection leading to speciation (Arnold et al., 2012; Whitney et al., 2010). Hybridization can occur between species of the same ploidy level (homoploid hybridization) and between species of different ploidy levels (heteroploid hybridization). In plants, hybridization with an increase in ploidy (allopolyploidy) is associated with speciation much more commonly than homoploid hybridization, partly because of reproductive isolation between hybrids and parents with different ploidy (Soltis et al., 2014; Soltis & Soltis, 2009). So far, only about 20 cases of homoploid hybrids have been well documented in plants (Gross & Rieseberg, 2005; White et al., 2018).

The Triticeae (Poaceae) is an important economic gene pool for genetic improvement of cereal and forage crops, including about 450 diploid and polyploid species distributed in a wide range of ecological habitats over the temperate, subtropical, and tropical pine regions (Dewey, 1984). The majority of species are allopolyploids, and the ploidy levels range from diploid (2n = 2x) to dodecaploid (2n = 12x). With combining a wide variety of biological mechanisms and genetic systems, the tribe Triticeae is an excellent group for research in evolution, genetic diversity, and speciation in plant polyploids (von Bothmer & Salomon, 1994; Paštová et al., 2019).

*Roegneria* C. Koch is a relatively large perennial genus in Triticeae, and includes approximately 130 species, most of which are tetraploid with the **StY** genome, nearly 70 of which are found in China (Yang et al., 2008). *Roegneria* species not only provided genetic material for the improvement of forage crops but could also be used as potential contributors of genes for cereal crops (Keng, 1959), such as *Roegneria stricta* Keng and *Roegneria turczaninovii* (Drob.) Nevski. Predecessors have reported some studies on the hybrids of *Roegneria*, such as a hybrid of *Roegneria* and *Hordeum* (Zhou et al., 1995), a hybrid of *R. ciliaris* and *Leymus multicaulis* (Zhang et al., 2008). These hybrids were created by the artificial hybridization and could not replace the value of natural hybrids.

Early identification of hybridization is mainly based on morphological characteristics. However, the reliability of morphological markers is low, and morphological intermediacy is not always related to hybridization. It may also be caused by convergent evolution or environment (Rieseberg, 1995). Cytological markers have been used as important evidence for hybridization, including karyotype analysis, meiotic pairing analysis, Genomic in situ hybridization (GISH), and Fluorescence in situ hybridization (FISH) (Han et al., 2004; Mao et al., 2017). However, due to the high parental chromosome homology of interspecific hybrids, it is difficult to explore origin of hybrids by FISH and GISH (Soltis et al., 1992). Single‐ or low‐copy nuclear genes, which are less susceptible to concerted evolution, can serve as useful markers for studies of phylogenetic relationships (Lei et al., 2018; Sha et al., 2010). DNA meiotic recombinase 1 (*DMC*1) gene has been used to examine hybridization events (Tang et al., 2017). The chloroplast DNA (cp DNA) is maternally inherited in grasses (Smith et al., 2006), and ribosomal protein S16 (*rps*16) is used to identify the maternal donor of genera in Triticeae (Yan et al., 2014).

**FIGURE 1:**
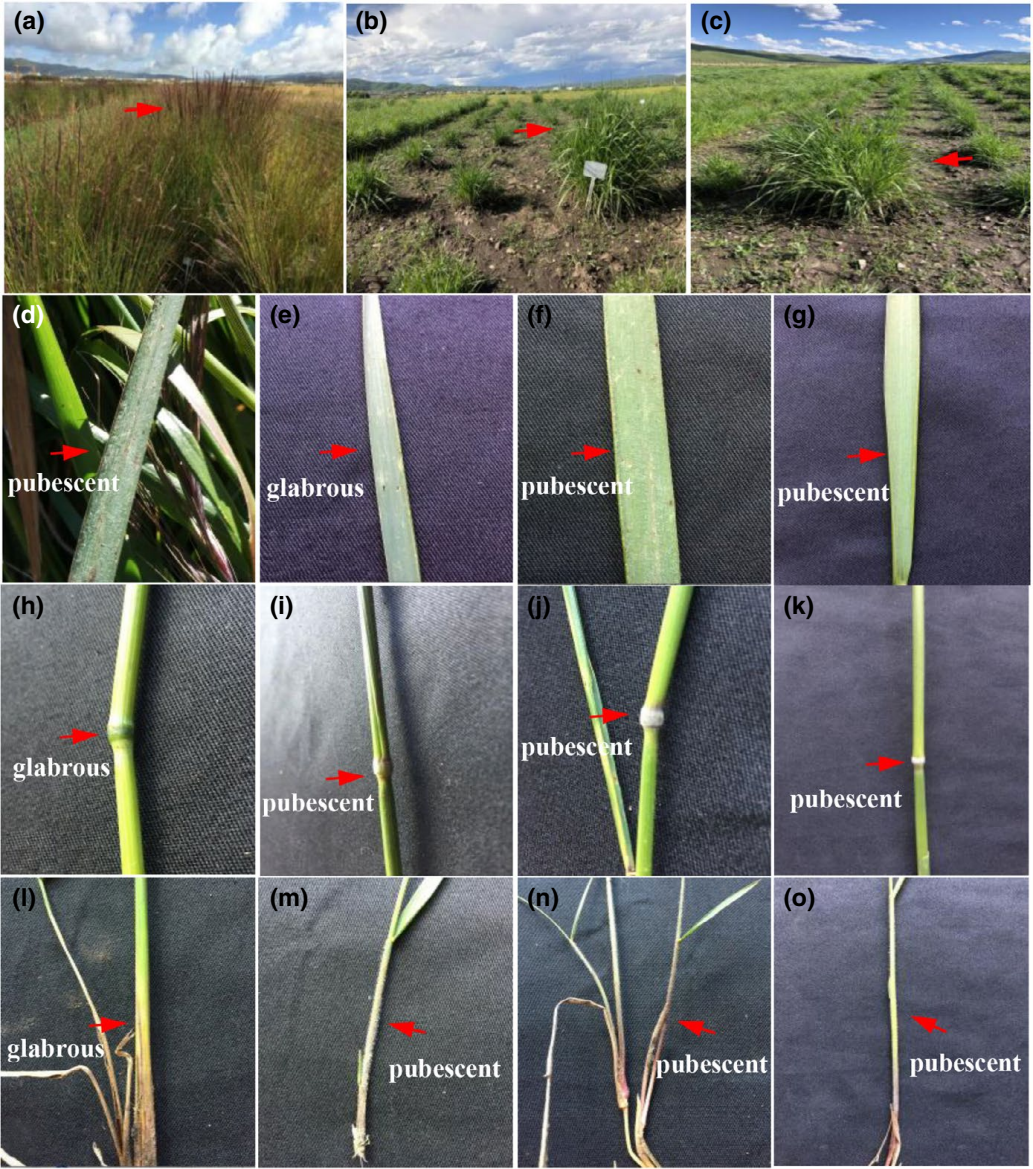
Morphological characteristics of the natural hybrids and their parents. (a) – (c) Natural distribution of hybrids. (a) hybrid RH1 (arrowed). (b) hybrid RH2 (arrowed). (c) hybrid RH1 (arrowed). (d–g) Leaves of hybrids and parents. (d) *R*. *turczaninovi*i (arrowed). (e) *R*. *stricta* (arrowed). (f) hybrid RH1 (arrowed). g: hybrid RH2 (arrowed). (h–k) Stem node of hybrids and parents. (h) *R*. *turczaninovi*i (arrowed). (i) *R*. *stricta* (arrowed). (j) hybrid RH1 (arrowed). k: hybrid RH2 (arrowed). (l–o) Basal leaves of hybrids and parents. (l) *R*. *turczaninovi*i (arrowed). (m) *R*. *stricta* (arrowed). (n) hybrid RH1 (arrowed). o: hybrid RH2 (arrowed)

To cultivate new forage varieties, *R*. *stricta* and *R*. *turczaninovii* cv. Linxi were planted very close in Hong yuan Research Base of the Sichuan Academy of Grassland Science (SAGS), Sichuan Province, China (31.47°N, 102.33°E). We harvested the seeds of the two species and planted them individually. In these plants, we found that some plants grew stronger and had lower seed setting rate than the surrounding plants (Figure 1a‐c), and they had intermediate morphological characters of *R*. *stricta* and *R*. *turczaninovii*, such as pubescence of leaf, basal leaf sheath, and stem node (Figure 1d‐o). We suspected that these plants are natural hybrids between *R*. *stricta* and *R*. *turczaninovii*. To determine if this is indeed the case, we conducted different methods including morphological analysis, cytological analysis, and phylogenetic analysis in these putative hybrids and their accompanying plants.

## 2 METHODS AND MATERIALS

### 2.1 Plant materials

Seventeen hybrids of RH1 (plants found in *R*. *stricta* field) and 40 hybrids of RH2 (plants found in *R*. *turczaninovii* field) were randomly distributed in fields. The possible parents *R*. *stricta* and *R*. *turczaninovii*, and the other Triticeae species growing nearby were also obtained, including *R*. *grandis* (**StY**), *E*. *sibiricus* (**StH**), and *Campeiostachys nutans* (**StYH**). All of them were collected from Hong Yuan Research Base of SAGS. Twenty diploid species (representing the genomes **St**, **H**, **E^e^**, **E^b^**, **W**, **P**, **Ta**, **V**, **Ns**, **A**, **B**, and **D**), *Roegneria* species (**StY**), *Elymus* species (**StH**), and *Campeiostachys* species (**StYH**) from the tribe Triticeae were used for cytological analysis and phylogenetic analysis. The names of the sampled taxa, abbreviations, accession numbers, ploidy level, genomic constitution, and GenBank accession numbers were listed in Table 1. Materials with PI and W_6_ were kindly provided by American National Plant Germplasm System (Pullman, WA, USA). The authors of this study collected all other accessions, for which voucher specimens were deposited with the perennial nursery and herbarium of the Triticeae Research Institute, Sichuan Agricultural University, China (SAUTI).

**TABLE 1:** Plant materials used in Phylogenetic analysis

Number	Species/hybrids	Genome	2n	Accession	Locality	GenBank No.	
						*DMC*1	*rps*16
1	RH1‐3	StY	4x	V 03	Sichuan, China	MZ130351*	MZ130373*
						MZ130352*	
2	RH1‐6	StY	4x	V 06	Sichuan, China	MZ130353*	MZ130374*
						MZ130354*	
3	RH1‐8	StY	4x	V 08	Sichuan, China	MZ130355*	MZ130375*
						MZ130356*	
4	RH1‐11	StY	4x	V 11	Sichuan, China	MZ130357*	MZ130376*
						MZ130358*	
5	RH1‐14	StY	4x	V 14	Sichuan, China	MZ130359*	MZ130377*
						MZ130360*	
6	RH2‐2	StY	4x	V 19	Sichuan, China	MZ130329*	MZ130362*
						MZ130330*	
7	RH2‐5	StY	4x	V 22	Sichuan, China	MZ130331*	MZ130363*
						MZ130332*	
8	RH2‐10	StY	4x	V 27	Sichuan, China	MZ130333*	MZ130364*
						MZ130334*	
9	RH2‐12	StY	4x	V 29	Sichuan, China	MZ130335*	MZ130365*
						MZ130336*	
10	RH2‐15	StY	4x	V 32	Sichuan, China	MZ130337*	MZ130366*
						MZ130338*	
11	RH2‐17	StY	4x	V 34	Sichuan, China	MZ130339*	MZ130367*
						MZ130340*	
12	RH2‐18	StY	4x	V 35	Sichuan, China	MZ130341*	MZ130368*
						MZ130342*	
13	RH2‐30	StY	4x	V 47	Sichuan, China	MZ130343*	MZ130369*
						MZ130344*	
14	RH2‐37	StY	4x	V 54	Sichuan, China	MZ130345*	MZ130370*
						MZ130346*	
15	RH2‐39	StY	4x	V 56	Sichuan, China	MZ130347*	MZ130371*
						MZ130348*	
16	*Roegneria strictus* (Keng) S.L. Chen	StY	4x	Y 2102	Sichuan, China	MZ130327*	MZ130361*
						MZ130350*	
17	*Roegneria turczaninovii* (Drobow) Nevski	StY	4x	ZY 11140	Inner Mongolia, China	MZ130328*	MZ130372*
						MZ130349*	
18	*Elymus sibiricus* L.	StH	4x	PI 619579	Xinjiang, China	EU366409*	
				BOP 022815		KP211332*	MK775250*
				PI 372541			
19	*Elymus caninus* L.	StH	4x	PI 314621	Former Soviet Union	EU366407*	
						EU366408*	
20	*Elymus elymoides* (Raf.) Swezey	StH	4x	PI 628684	United States	FJ695161*	
						FJ695160*	
21	*Elymus glaucus* Buckley	StH	4x	PI 593652	Oregon United States	FJ695163*	
						FJ695162*	
22	*Elymus virginicus* L.	StH	4x	PI 490361	United States	GQ855195*	
				PI 882397	Sichuan, China	GQ855196*	
23	*Elymus wawawaiensi*	StH	4x	PI 506284	Sichuan, China		
24	*Roegneria caucasica* K. Koch	StY	4x	H 3207	Xinjiang, Armenia	HM770785*	
						HM770784*	
25	*Roegneria ciliaris* (Trin.) Nevski	StY	4x	87‐88 335	Sichuan, China	KU160610*	
				88‐89‐238		KU160617*	
26	*Roegneria dura* Keng	StY	4x	Y 2124	Neimenggu, China	KX578879*	
27	*Roegneria grandis* Keng	StY	4x	ZY 3189	Xizang, China	KU160615*	MN703669*
				Y 3189		KU160618*	
28	*Roegneria hondai* Kitagawa	StY	4x	Y 0362	Sichuan, China	KX578840*	
						KX578841*	
29	*Roegneria longearistata* (Boiss.) Drob.	StY	4x	Y 2259	Inner Mongolia, China	KX578848	
30	*Roegneria shandongensis* (B. Salomon) J. L. Yang & C. Yen	StY	4x	ZY 3150	Shanxi, China	KX578862*	
31	*Roegneria ugamica* (Drob.) Nevski	StY	4x	Y 1698	Sichuan, China	KX578877*	
						KX578878*	
32	*Campeiostachys nutans* (Griseb.) J. L. Yang, B. R. Baum et C. Yen	StYH	6x	Y 2086	Sichuan, China		
				ZY 17101		KX578851*	
				ZY 17102		KX578852*	MT385866*
				S 22‐4		KX578853*	
33	*Pseudoroegneria libanotica* (Hackel) D. R. Dewey	St	2x	PI 228389	Iran	FJ695174*	
				PI 228392			
34	*Pseudoroegneria spicata* (Pursh) A. Löve	St	2x	PI 547161	United States	FJ695175*	KY636118*
				PI 632532			
35	*Pseudoroegneria stipifolia* (Czern. ex Nevski)	St	2x	PI 325181	Stavropol, Russian	FJ695176*	
36	*Pseudoroegneria strigosa* (M. Bieb.) A. Löve	St	2x	PI 595164	Xinjiang, China	FJ695177*	
				PI 499637			
37	*Pseudoroegneria tauri* (Boiss.) A. Löve	St	2x	PI 401329	Iran	KU160613	
				PI 380650			
38	*Agropyron cristatus* (L.) Gaertn	P	2x	H 4349	China	AF277241*	KY126307*
				PI 598628	Kazakhstan		
39	*Australopyrum retrofractum* (Vickery) A. Löve	W	2x	H 6723	China	AF277251*	KY636080*
				PI 531553	United States		
40	*Hordeum chilense* Roem. & Schult.	H	2x	PI 531781	Chile	FJ695173*	
41	*Hordeum pubiflorum* Hook. f.	H	2x	BCC 2028			KY636108*
42	*Hordeum bogdanii* Wilensky	H	2x	PI 531761	China	FJ695172*	MH331641*
43	*Hordeum vulgare* L.	I	2x	H 3878	Italy		EF115541*
44	*Lophopyrum elongatum* (Host) A. Löve	E^e^	2x	PI 531719	Israel	AF277246*	
				PI531718			MH331643*
45	*Thinopyrum bessarabicum* (Savul. & Rayss) A.	E^b^	2x	PI 531711	Russia	AF277254*	KY636145*
				W6 21890			
46	*Psathyrostachys huashanica* Keng ex P.C Kuo	Ns	2x	PI 531823	Shanxi, China	GU165826*	
47	*Aegilops speltoides* Tausch.	B	2x	H 6779		DQ247833*	
48	*Aegilops tauschii* Coss.	D	2x	H 6668		AF277235*	
				AE 429			JQ754651*
49	*Dasypyrum villosum* (K. Koch) Nevski	V	2x	H 5552		AF277236*	
				W6 7264			MH285850*
50	*Secale cereale* L.	R	2x				KC912691*
51	*Taeniatherum copmedusae* (L.) Nevski	Ta	2x	H 10254		AF277249*	
				PI 220591			MH285856*
52	*Triticum urartu* Tum.	A	2x	H 6664		DQ247826*	
53	*Bromus sterilis* L.			OSA 420		AF277234*	

Note

1* Data from published sequences in the GenBank (http://www.ncbi.nlm.nih.gov).

### 2.2 Morphological analysis

Morphology among plants of putative hybrids, *R*. *stricta*, *R*. *turczaninovii*, *R*. *grandis*, *E*. *sibiricus*, and *C*. *nutans*, was measured for 21 characters. The Euclidean distance was calculated by the dist function in R. The hclust function in R was used to cluster. The tree was plotted by ggtree package in R.

### 2.3 Pollen fertility and seed set

The pollen grains from mature anthers were stained in an I_2_‐KI solution for pollen fertility study. Seed set was estimated from a 10‐spike sample per plant.

### 2.4 Karyotype and meiotic pairing analysis

Karyotype analysis was followed by Gill et al. (1991). The procedures of fixation, staining, and calculation of meiotic pairing followed Zhang and Zhou (2006).

### 2.5 Chromosome preparation and *in situ* hybridization

Chromosomes were prepared for GISH analysis according to the method of Han et al. (2004). Total genomic DNA was extracted from fresh leaves by the CTAB method (Murray & Thompson, 1980). Plasmids (from positive clones that are **St** genome) and the **StY** genome were labeled with fluorescein‐12‐dUTP or Texas‐red‐5‐dCTP using the nick translation method. Hybridization procedure, detection, and visualization were performed according to the method of Wang et al. (2017).

### 2.6 Amplification and sequencing

The *DMC*1 and *rps*16 gene were amplified using the primers listed in Table S1 (Petersen & Seberg, 2002; Shaw et al., 2005). All PCRs were conducted in a 50‐μl reaction volume, with 1.5 U Ex Taq polymerase (TaKaRa, Shiga, Japan). The PCR amplification protocols for the *DMC*1 and *rps*16 gene are presented in Table S1. PCR products were cloned into the pMD19‐T vector (TaKaRa). At least 15 random independent clones were selected for sequencing by Shanghai Sangon Biological Engineering and Technology Service Ltd. (Shanghai, China).

### 2.7 Phylogenetic analysis

DNA sequences were confirmed through BLAST nucleotide alignment in the NCBI database, and sequence alignments were made using MAFFT (Katoh & Standley, 2013). After preliminary phylogenetic analysis, the number of sequences is reduced. If there are more sequences of the same species form monophyletic groups, only one sequence is retained. ModelTest v3.06 (Posada & Crandall, 1998) was used to determine appropriate DNA substitution models and gamma rate heterogeneity using the Akaike information criterion (AIC).

The phylogenetic analyses of *DMC*1 and *rps*16 data were performed by using the maximum‐likelihood (ML) method in PhyML 3.0 (Guindon et al., 2009). The best‐fit evolutionary models determined were TPM1uf+G for *DMC*1 and TIM1+G for rps16. As a measurement of the robustness of tree clades, the bootstrap support (BS) values were calculated with 1000 replications and displayed in figure (above the branch) if the BS values were >50% (Felsenstein, 1985). Bayesian analyses were also performed using MrBayes 3.1 (Ronquist & Huelsenbeck, 2003). The evolutionary model selected default settings.

## 3 RESULTS

### 3.1 Morphological characteristics

The 57 natural hybrids were perennial grasses, which were similar in morphology and phenology to *Roegneria* species, such as one spikelet per node and palea equaling lemma. Most of hybrids were stronger than their surrounding plants (Figure 1a–c). These natural hybrids combined some unique characteristics of *R*. *stricta* and *R*. *turczaninovii*, such as leaf pubescence, stem node pubescence, and basal leaf sheath pubescence (Figure 1d‐o).

**FIGURE 2:**
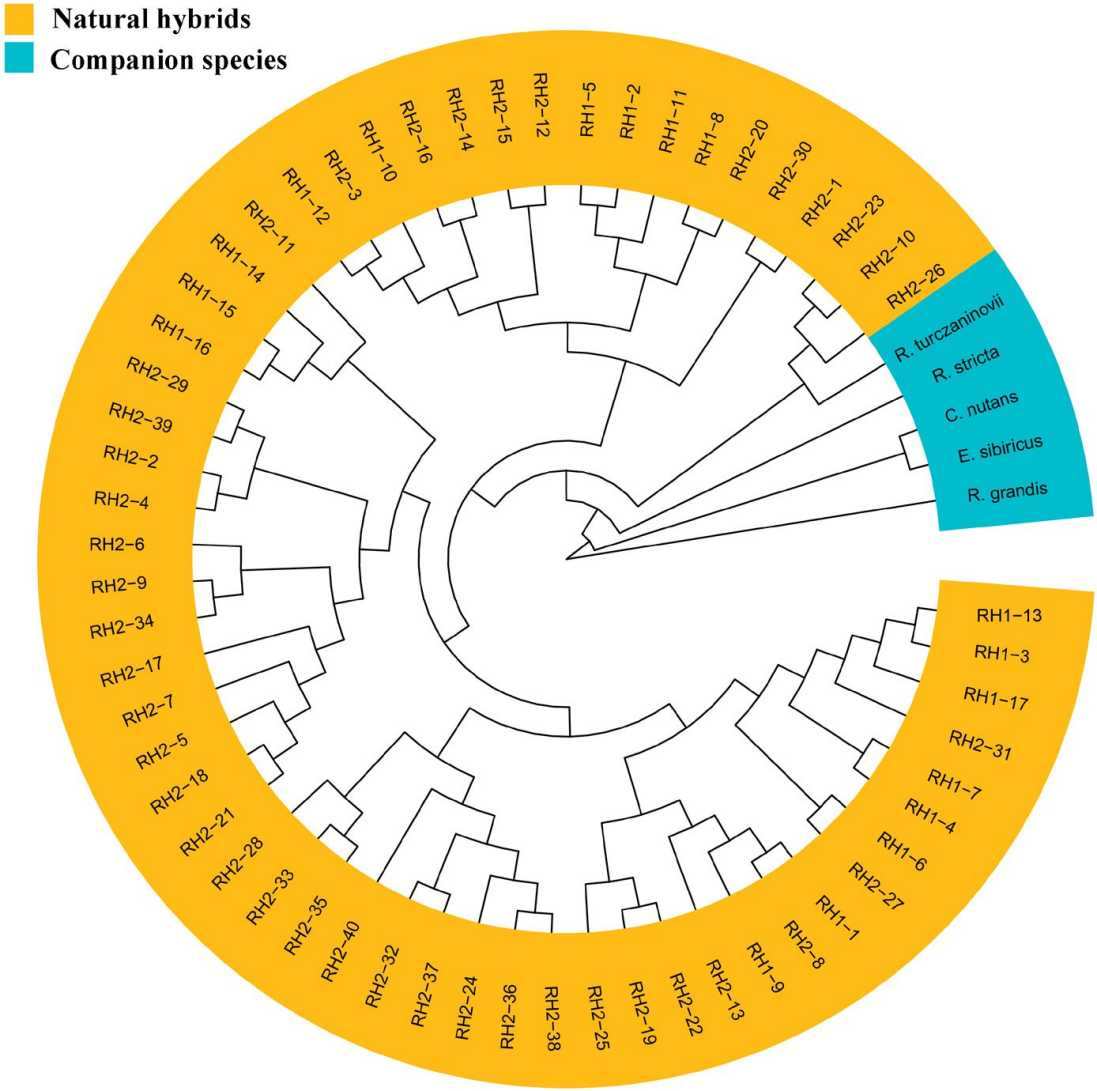
Cluster analysis of hybrids RH1, hybrids RH2, *R*. *stricta*, *R*. *turczaninovii*, *R*. *grandis*, *E*. *sibiricus* and *C*. *nutans* based on 21 morphological characters. Morphology including Top internodes length, First lemma length, Palea length, First glume length, First glume width, Second glume length, Second glume width, Flag leaf length and width, Top second leaf length and width, Spike length, Plant height, Awn length of first lemma, No. of spikelets per spike, No. of florets per spikelet, Hair on sheath, Hair on stem node, Hair on leaf, Awn

Morphology among plants of putative hybrids, *R*. *stricta*, *R*. *turczaninovii*, *R*. *grandis*, *E*. *sibiricus*, and *C*. *nutans*, was measured for 21 characters. Cluster analysis based on 21 morphological characters was shown in Figure 2. The results of cluster analysis indicated that 57 natural hybrids were closer to *R*. *stricta* and *R*. *turczaninovii*.

### 3.2 Evaluation of pollen fertility and seed set

**FIGURE 3:**
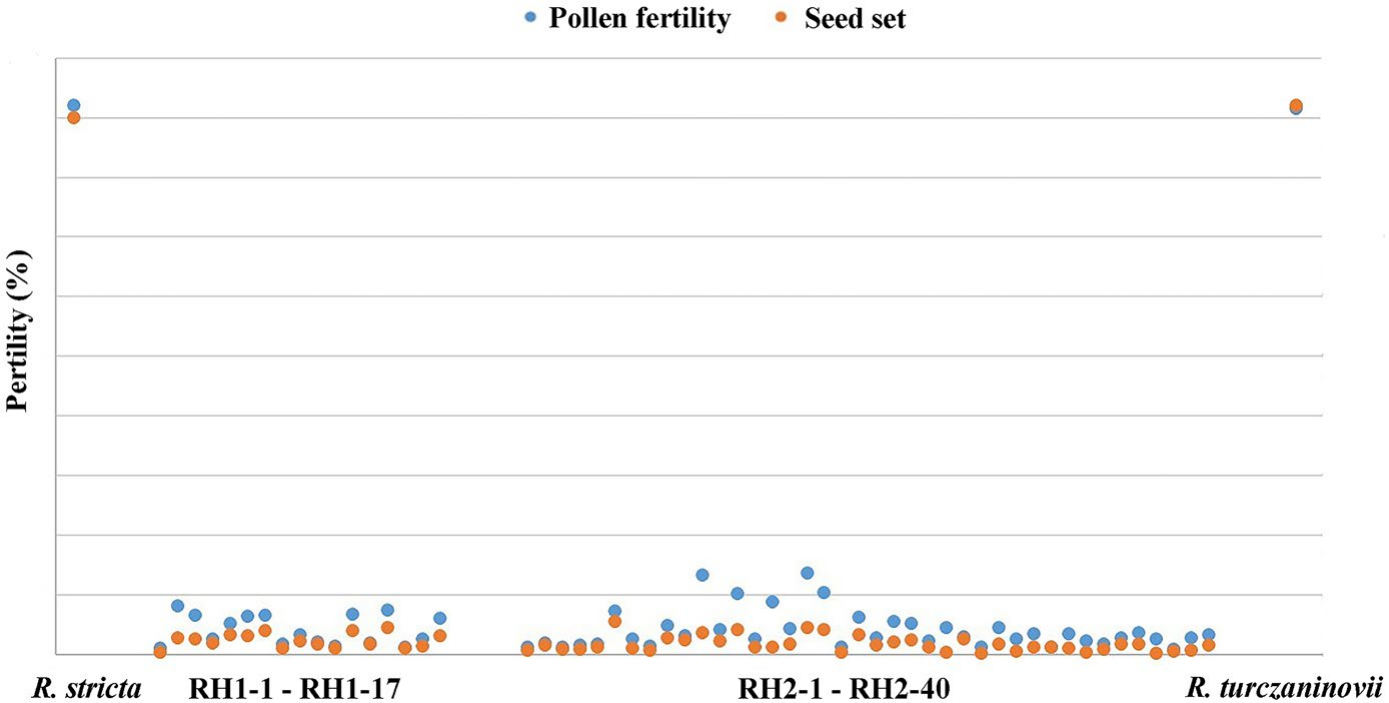
Pollen fertility and seed set of hybrids RH1, hybrids RH2, *R*. *stricta* and *R*. *turczaninovii*

The fertility, including pollen fertility and seed set, of *R*. *stricta*, *R*. *turczaninovii*, and putative hybrids, was shown in Figure 3. In *R*. *stricta*, the pollen fertilities were up to 92.05% and the seed sets were 90.02%. In *R*. *turczaninovii*, the pollen fertilities and seed set were high with 91.61% and 92.18%, respectively.

As for the hybrids of RH1, the pollen fertilities varied from 1.01% to 8.09%, and the seed sets were lower than those of their possible parents, varying from 0.41% to 4.50% (Figure 3). As for the hybrids of RH2, the pollen fertilities varied from 0.83% to 13.63%, and seed set were lower, varying from 0.23% to 5.59% (Figure 3). It could be seen that the pollen fertilities and seed sets of putative hybrids were very low, indicating that they were hybrids and not stable species.

### 3.3 Karyotype analysis and chromosome pairing at metaphase I

**FIGURE 4:**
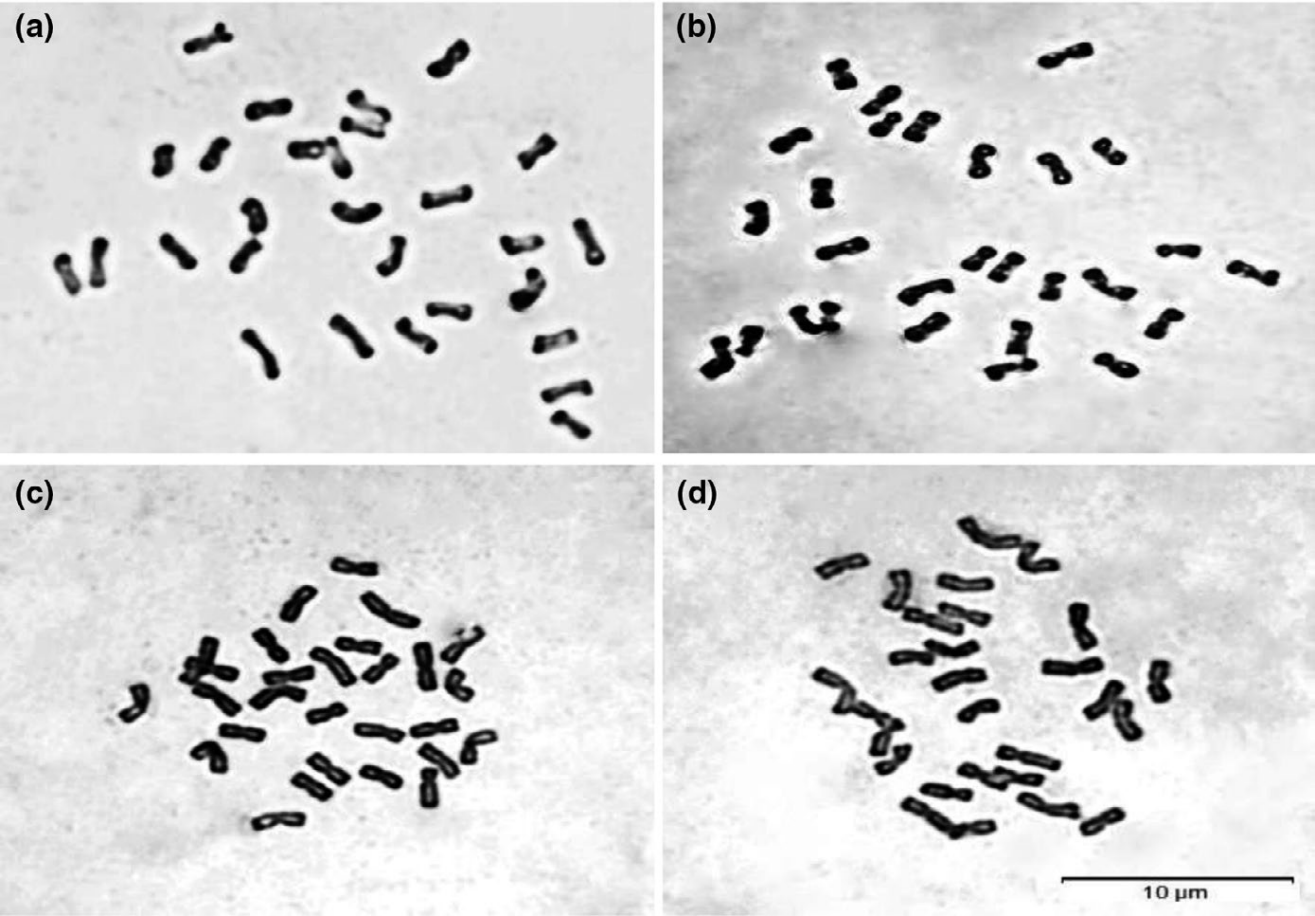
Karyotype analysis of hybrids RH1, hybrids RH2, *R*. *stricta* and *R*. *turczaninovii*. (a) *R*. *turczaninovii*. (b) *R*. *stricta*. (c) RH1‐13. (b) RH2‐10

Karyotype analysis showed that *R*. *stricta*, *R*. *turczaninovii*, and putative hybrids were tetraploids (2n = 4x = 28) (Figure 4).

**FIGURE 5:**
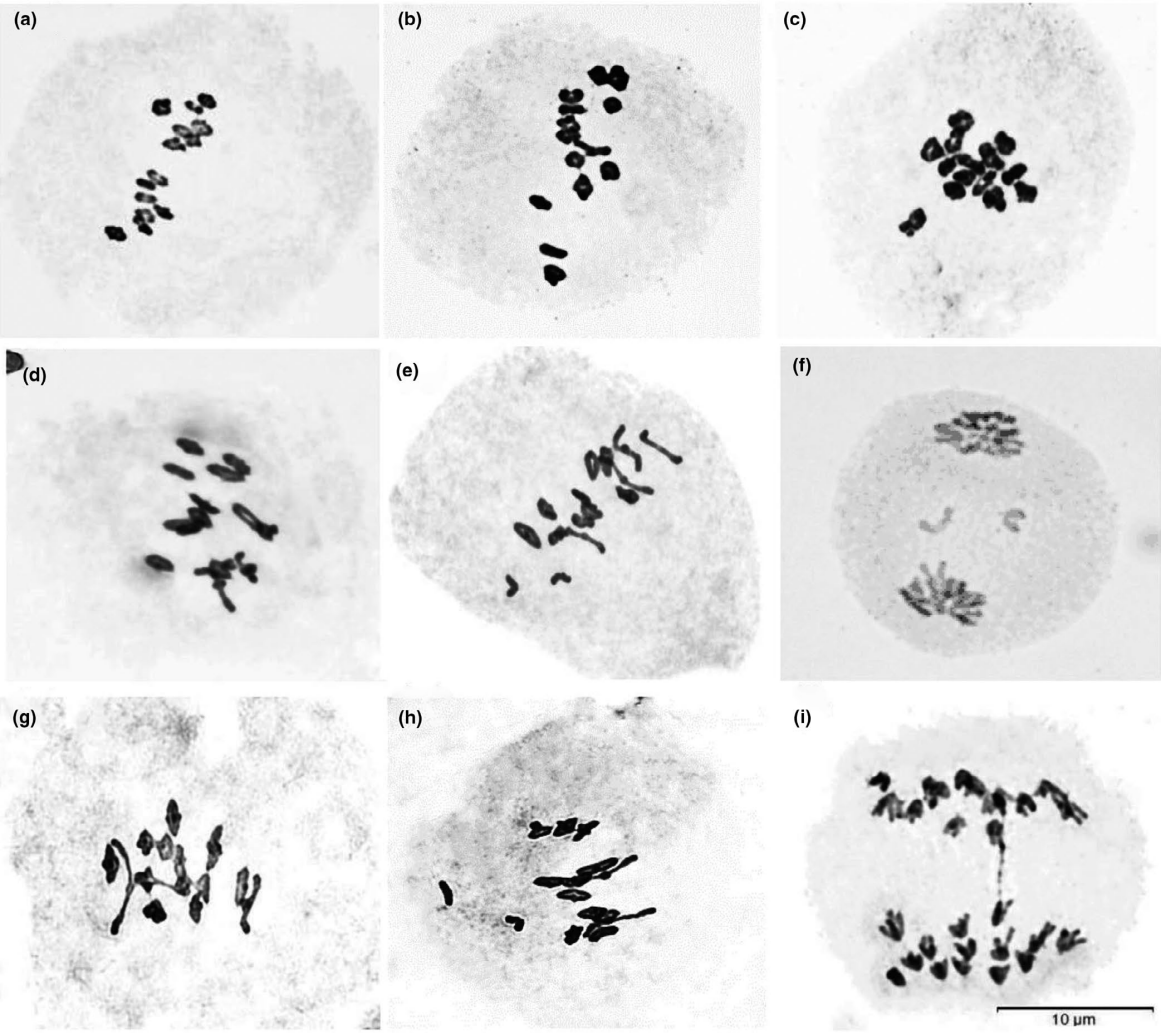
Meiotic associations in PMCs of the parental species and hybrids. (a) and (b) *R*. *turczaninovii* with 14 II. (c) *R*. *stricta* with 14 II. (d) RH1‐15 with 14 II (12 ring + 2 rod). (e) RH1‐7 with 13 II (9 ring + 8 rod) + 2 I. (f) RH2‐38 lagging chromosomes. (g) RH2‐29 with 14 II (12 ring and 2 rod). (h) RH2‐11 with 13 II (11 ring and 2 rod) + 2 I. (i) RH2‐11 chromosome bridge

The meiotic configurations of the possible parent and the putative hybrids were listed in Table S2. Meiosis of *R*. *stricta* and *R*. *turczaninovii* were quite regular with 14 bivalents (Figure 5a–c, Table S2). Meiotic pairing in 17 hybrids of RH1 was comparatively high, with an average of 0.98 univalents and 13.52 bivalents per cell with c‐value of 0.89 (Figure 5d, e; Table S2). Chromosome pairing in 40 hybrids of RH2 was comparatively high with an average of 0.85 univalents and 13.55 bivalents per cell with *c*‐value of 0.90 (Figure 5g, h; Table S2). Except for hybrid RH2‐31, all hybrids had univalents.

At the same time, some lagging chromosomes and chromosome bridges were observed at anaphase I (Figure 5f, i).

### 3.4 FISH and GISH analysis

To further explore the genomic constitutions of natural hybrids, we selected some hybrids for *in situ* hybridization. Since the suspected parents of natural hybrids were *R*. *turczaninovii* and *R*. *stricta* (**StY**), and meiotic pairing in natural hybrids were comparatively high, we speculated that genomic constitution of natural hybrids was **StY**. St_2_‐80 was a FISH marker for the **St** genome (Wang et al., 2017). Signals produced by St_2_‐80 were present on the entire arm of the **St** genome chromosomes, except at the centromeric region and near centromeric region (Wang et al., 2017). This marker was used to detect the **St** genome presented in the putative parents and hybrids.

**FIGURE 6:**
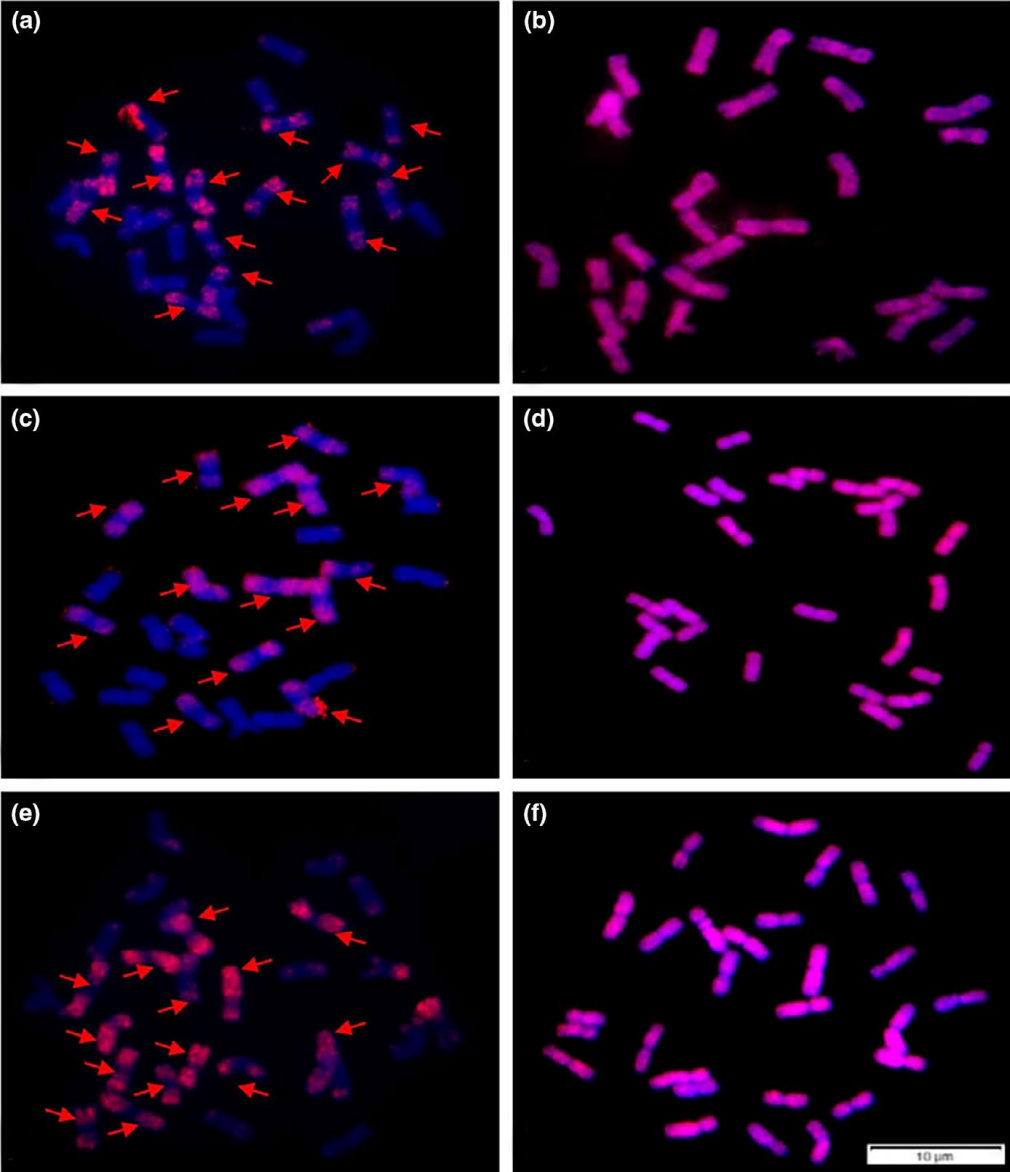
Analysis of FISH and GISH in *R*. *stricta* and *R*. *turczaninovii*. (a) and (b) *R*. *stricta*. (c–f) *R*. *turczaninovii*. (a), (c) and (e) Used St_2_–80 as probe (red), 14 chromosomes were labeled as **St** type (arrowed) and 14 chromosomes were labeled as non‐St type. (b), (d) and (f) With total genomic DNA of *R*. *ciliaris* (**StY** genome) was labeled with Texas‐red‐5‐dCTP (red) as probe, 28 chromosomes were labeled as red fluorescent signals

**FIGURE 7:**
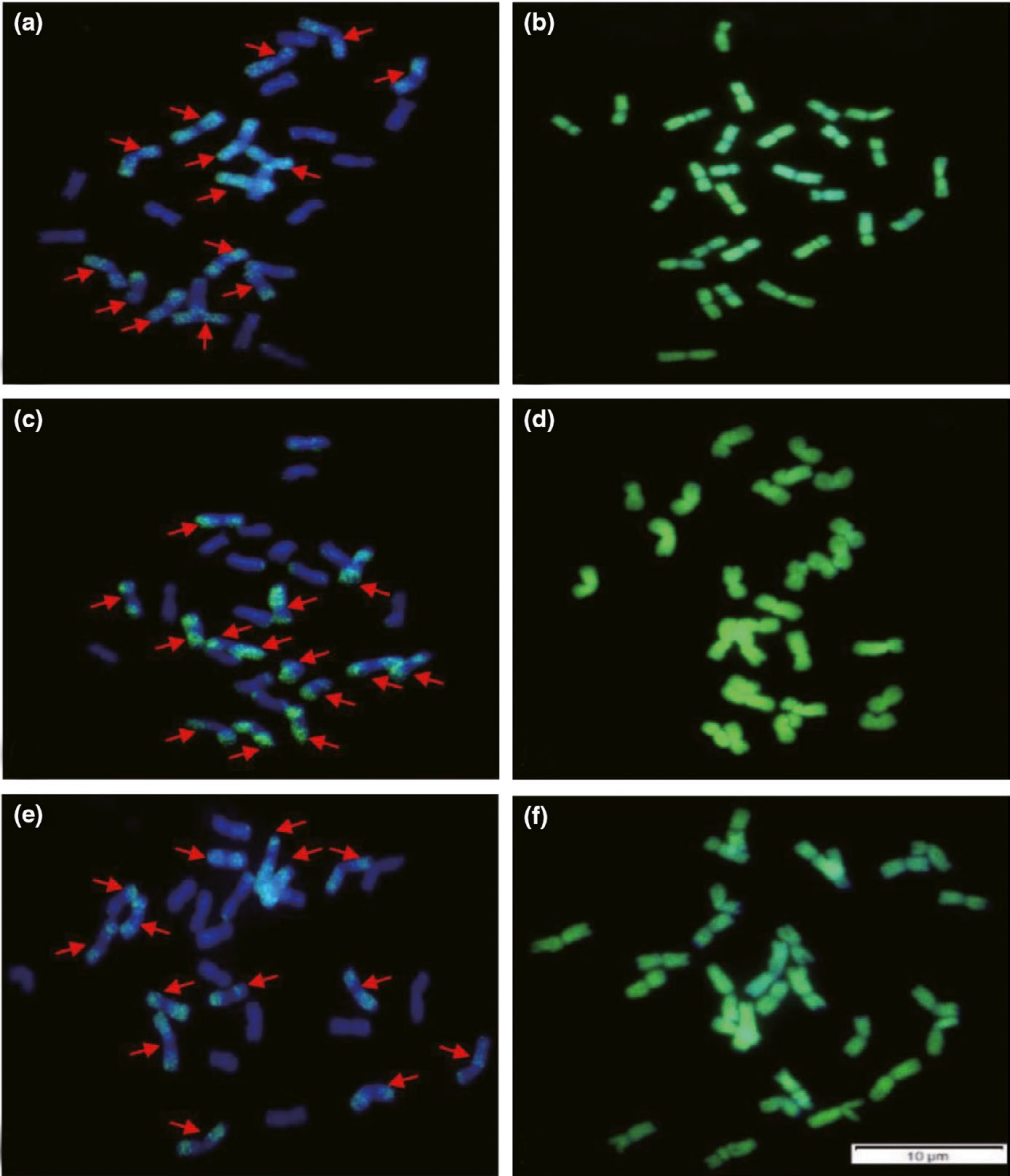
Analysis of FISH and GISH in hybrids. (a) and (b) RH1‐11. (c) and (d) RH2‐12. E and F RH2‐15. (a), (c), and (e) Using St_2_–80 as probe (green), 14 chromosomes were labeled as **St** type (arrowed) and 14 chromosomes were labeled as non‐St type. (b), (d) and (f) With total genomic DNA of *R*. *ciliaris* (**StY** genome) was labeled with fluorescein‐12‐dUTP (green) as probe, 28 chromosomes were labeled as green fluorescent signals

St_2_‐80 signal pattern showed that 14 chromosomes of putative parents and hybrids were **St** type (Figures 6a, c, e and 7a, c, e). This result was confirmed by GISH analysis, where 28 chromosomes of putative parents and hybrids were hybridized with the **StY** probe from *R*. *ciliaris* (Figures 6b, d, f and 7b, d, f). The results of FISH and GISH indicated that the genomic constitution of putative parents and 11 hybrids (RH1‐3, RH1‐8, RH1‐11, RH1‐14, RH2‐2, RH2‐10, RH2‐12, RH2‐15, RH2‐17, RH2‐37, RH2‐39) was **StY**.

### 3.5 Phylogenetic analyses of the nuclear gene *DMC*1 and the chloroplast gene *rps*16 sequences

**FIGURE 8:**
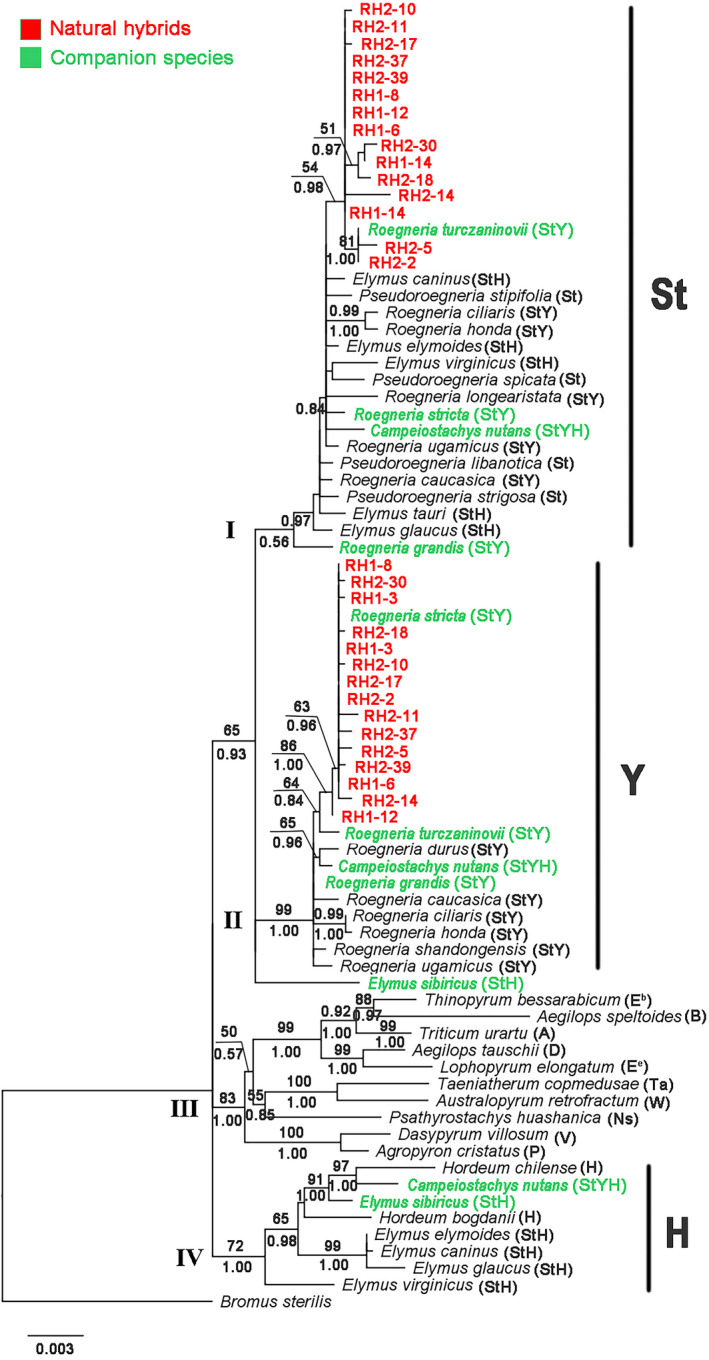
Phylogenetic tree based on *DMC*1 sequences of hybrids using ML. Numbers with bold above nodes are bootstrap values, and numbers below nodes are Bayesian posterior probability values

In order to analyze the possible parents of the hybrids, we analyzed the nuclear gene *DMC*1 and the chloroplast gene *rps*16 sequences of the hybrids and their associated species of *Roegneria*, *Elymus*, and *Campeiostachys*. The length of *DMC*1 sequences of hybrids ranged from 998 to 1004 bp. The data matrix contained 1166 characters, of which 267 characters were variable and 235 were parsimony informative. A single phylogenetic tree generated by maximum likelihood analysis using the TPM1uf + G model (−Ln likelihood = 4762.04) was shown in Figure 8.

The phylogenetic analyses of the *DMC*1 sequence were shown in Figure 8. In clade I (PP = 0.97), the **St**‐type sequences formed a strongly supported clade, which included diploid *Pseudoroegneria* (**St**) species, tetraploid E*lymus* (**StH**) and *Roegneria* (**StY**) species, hexaploid *campeiostachys* (**StYH**) species, and hybrids. The **St**‐type sequences of 15 hybrids and *R*. *turczaninovii* (**StY**) formed a subclade (BS = 54%, PP = 0.98). In clade Ⅱ (BS = 99%, PP = 1.00), the **Y**‐type sequences formed a strongly supported clade, which contained the tetraploid species of *Roegneria* (**StY**) and hybrids. The **Y**‐type sequences of 15 hybrids, *R*. *turczaninovii* (**StY**) and *R*. *stricta* (**StY**), formed a subclade (BS = 64%, PP = 0.84). In clade Ⅲ (BS = 83%, PP = 1.00), 10 diploid species contained 10 different basic genomes (**E^e^**, **E^b^**, **W**, **P**, **Ta**, **V**, **Ns**, **A**, **B**, and **D**). In clade IV (BS = 72%, PP = 1.00), the **H**‐type subclade included diploid *Hordeum* species and tetraploid *Elymus* (**StH**) species.

**FIGURE 9:**
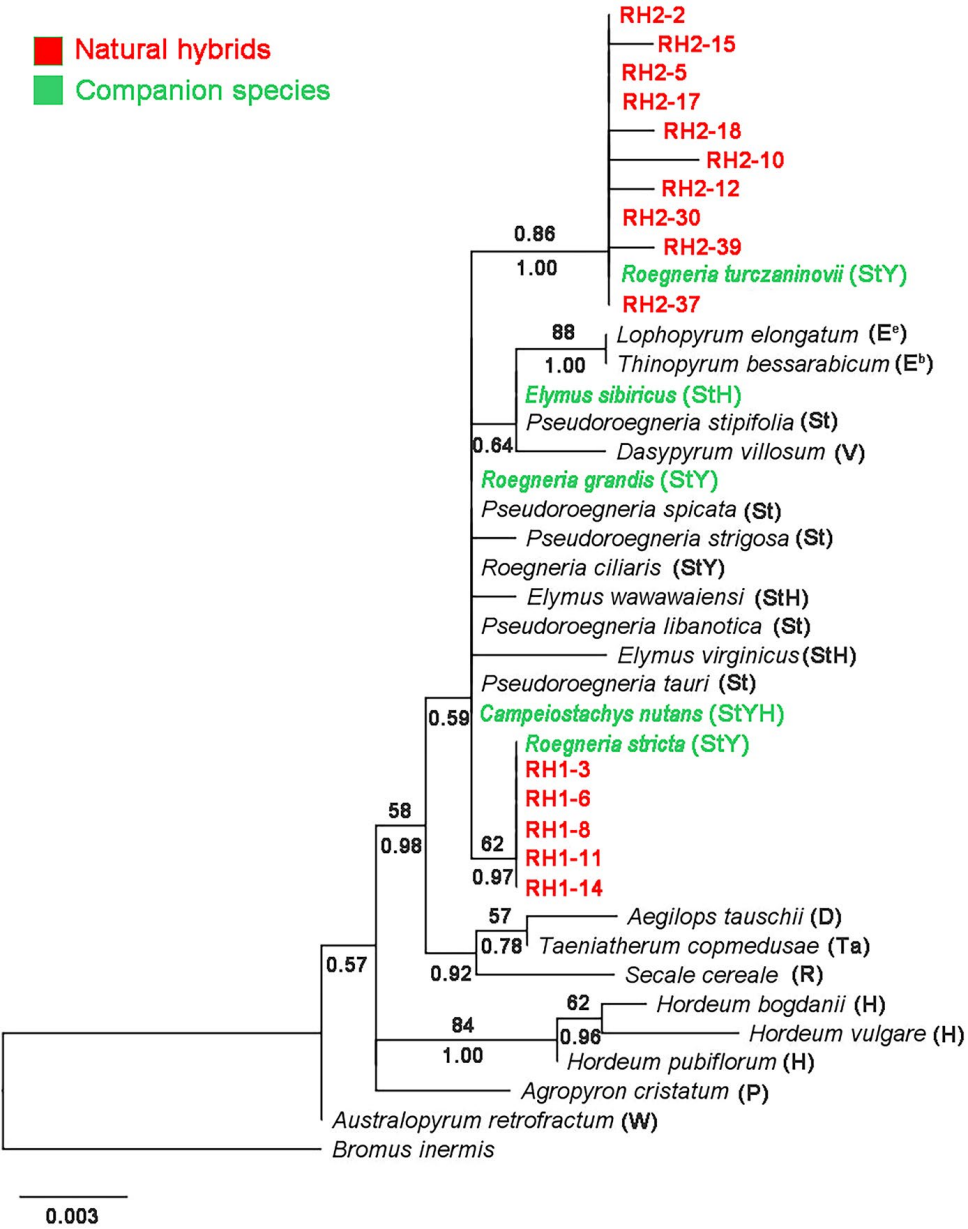
Phylogenetic tree based on *rps*16 sequences of hybrids using ML. Numbers with bold above nodes are bootstrap values, and numbers below nodes are Bayesian posterior probability values

The length of hybrids of *rps*16 sequences varied from 830 to 831 bp. The data matrix contained 881 characters, of which 30 were variable characters and 30 were parsimony informative. TIM1 + G as the best‐fit model (−Ln likelihood = 1550.15) was used in phylogenetic analysis. The ML tree was displayed in Figure 9.

The phylogenetic analyses of the *rps*16 sequence were shown in Figure 9. The *rps*16 sequences from hybrids of RH1 were grouped with *R*. *stricta* (BS = 62%, PP = 0.97). This clade contained 5 hybrids of RH1 sequences and *R*. *stricta*. The *rps*16 sequences from hybrids of RH2 were grouped with *R*. *turczaninovii* (BS = 86%, PP = 1.00). This clade contained 10 hybrids of RH2 sequences and *R*. *turczaninovii*. The above results showed that *R*. *stricta* was the maternal donor of the hybrids of RH1, while *R*. *turczaninovii* was the maternal donor of the hybrids of RH2.

## 4 DISCUSSION

### 4.1 Origin of natural hybrids

Natural hybrids are relatively common in flowering plants (Rieseberg & Ellstrand, 1993). Rieseberg (1997) reported that about 11% of plant species arose from interspecific hybridization. Artificial hybrids involving genus *Roegeneria* have been produced (Zhou et al., 1999), but there are no reports of natural hybrids. In this study, the low‐fertility plants were suspected natural hybrids because of their morphologically intermediate between *R*. *stricta* and *R*. *turczaninovii*. However, the natural hybrids had not been confirmed by cytological and molecular evidence. In this study, FISH and GISH analysis suggested that the genomic constitution of *R*. *turczaninovii* was **StY**. This result was further confirmed by molecular data. Phylogenetic analyses based on *DMC*1 sequence suggested that *R*. *turczaninovii* has **St** and **Y** genomes. It is the first report that the composition of the genomic formula of *R*. *turczaninovii* is **StY**, confirming that the current taxonomic status is correct. The natural hybrids were verified unambiguously because of morphological characteristics, and molecular sequences of natural hybrids were closer to those of *R*. *stricta* and *R*. *turczaninovii* in companion species (Figures 2 and 8). Phylogenetic analysis based on *rps*16 sequence showed that *R*. *stricta* was the maternal donor of the hybrids of RH1, *R*. *turczaninovii* was the maternal donor of the hybrids of RH2 (Figure 9). Thus, our results demonstrated that *R*. *stricta* and *R*. *turczaninovii* were the female and male parents, respectively, of the hybrids of RH1; *R*. *turczaninovii* and *R*. *stricta* were the female and male parents of the hybrids of RH2, respectively.

Additionally, meiotic pairing in 57 natural hybrids was comparatively high. This suggested that the genomes of their parents were homologous. This is consistent with our cytology and molecular data. Except for hybrid RH2‐31, all hybrids had univalent. This also provides evidence for the low pollen fertility and seed setting rate of hybrids. Pairing and recombination among homologous chromosomes are common in nascent allopolyploids (Gaeta & Pires, 2010). However, in the evolution of allopolyploids, homologous pairing is gradually eliminated and replaced by exclusive homologous pairing. *R*. *stricta* and *R*. *turczaninovii* contain the **StY** genome, but the genomes may have diverged in the two species, resulting in hybrids showing univalent at metaphase I. The chromosome bridge appeared to be in some natural hybrids at anaphase (Figure 5i). Such chromosome bridges might be formed by single‐ or three‐strand doubles within the reverse loop of a paracentric inversion heterozygote, and the chromosome bridge was a sign of inversion; these were important events in speciation.

### 4.2 Formation process of natural hybrids

Triticeae is a young group; there is a large possibility of random hybridization among the relative genera in the Triticeae (Barkwoth & Bothmer, 2009). In this study, different genera species with different genome constitutions in Triticeae were planted in the experiment base of the SAGS, such as *Roegneria* (**StY**), *Elymus* (**StH**), and *Campeiostachys* (**StYH**). *R*. *stricta* and *R*. *turczaninovii* have closer genetic relationship, the florescence was consistent, and they were planted together, which provided conditions for natural hybridization.

From the perspective of hybridization rate, there were 23 hybrids out of which about 400 were *R*. *stricta* plants, and the natural hybridization rate was about 5.75%, while among the 330 *R*. *turczaninovii* plants, there were about 54 hybrids, and natural hybridization rate was about 16.36%. It can be seen that natural hybridization rate of *R*. *turczaninovii* was about 3 times that of *R*. *stricta*. The reason may be that the source of the *R*. *stricta* parents was single and the genetic diversity was low, while the *R*. *turczaninovii* parent has higher genetic diversity. Large morphological differences were observed in the field of *R*. *turczaninovii*, which lead to a higher natural hybridization rate. The genetic diversity of the *R*. *stricta* parents and *R*. *turczaninovii* parents needed to be further verified by molecular markers or other methods.

### 4.3 Homoploid hybrid speciation

In the evolutionary history, many grasses from the Triticeae have undergone interspecific hybridization, resulting in allopolyploidy, which homoploid hybrid speciation (HHS) was found only in rye (Martis et al., 2013). Homoploid hybrid speciation is rare due to strongly reduced fitness of early generation hybrids and weak reproductive isolation with the progenitors (Mallet, 2007; Rieseberg & Willis, 2007). Our comprehensive analyses of natural hybrids, *R*. *stricta*, *R*. *turczaninovii* and the other Triticeae species growing nearby from morphology, cytology, and molecular levels provided support for the origin of natural hybrids. It demonstrates for the first time the existence of populations of natural homoploid hybrids in *Roegneria*. Analyses of hybrid swarms or young hybrid taxa can play an important role in elucidating the first steps toward hybrid species (Nolte & Tautz, 2010). Although such taxa may not eventually produce well‐differentiated hybrid species, they can facilitate testing key predictions from models of hybridization and hybrid speciation (Barton, 2001; Buerkle et al., 2000). In this study, the natural homoploid hybrids are good research material for elucidating the first steps toward homoploid hybrids species. They can facilitate testing of key predictions from hybridization and hybrid speciation models. It can provide some references for the formation mechanism of natural hybrids of Triticeae.

### 4.4 Utilization of natural hybrids

Hybridization among species can act as an additional, perhaps more abundant, source of adaptive genetic variation than mutation (Arnold & Martin, 2009; Kunte et al., 2011; Whitney et al., 2010). In this study, we found some natural hybrids with good forage traits in plant height, tillers, and leaf, but the fertility was very low. If these natural hybrids could be genetically improved to create new forage varieties, it would have good ecological and economic benefits. As a result of further reproduction, these hybrids could be a valid species because some highly sterile F_1_ hybrids become species through adopting a vegetative mode of reproduction (Brysting et al., 2000).

## CONFLICT OF INTEREST

The authors declare no conflict of interest.

## AUTHOR CONTRIBUTION

**Chen Chen:** Conceptualization (equal); Formal analysis (lead); Investigation (lead); Methodology (equal); Project administration (equal); Writing – original draft (lead); Writing – review & editing (equal). **Zilue Zheng:** Conceptualization (equal); Data curation (equal); Formal analysis (equal); Investigation (equal); Software (lead); Writing – original draft (equal); Writing – review & editing (equal). **Dandan Wu:** Conceptualization (equal); Data curation (equal); Formal analysis (equal); Resources (supporting); Software (equal); Supervision (equal); Visualization (equal). **Lu Tan:** Formal analysis (equal); Investigation (supporting); Software (equal). **Cairong Yang:** Conceptualization (supporting); Methodology (supporting). **Songqing Liu:** Resources (supporting). **Jiale Lu:** Project administration (supporting); Supervision (supporting); Validation (supporting). **Yiran Cheng:** Formal analysis (supporting); Methodology (supporting); Software (supporting). **Lina Sha:** Visualization (supporting). **Yi Wang:** Validation (supporting). **Houyang Kang:** Supervision (supporting). **Xing Fan:** Software (supporting). **Yonghong Zhou:** Validation (supporting). **Changbing Zhang:** Data curation (equal); Project administration (equal); Resources (equal); Supervision (equal). **Haiqin Zhang:** Conceptualization (equal); Funding acquisition (lead); Investigation (equal); Project administration (equal); Resources (equal); Supervision (lead); Writing – original draft (equal); Writing – review & editing (equal).

## Supporting information

Table S1‐S2

## Data Availability

Morphological data are available from the Dryad Digital Repository at https://doi.org/10.5061/dryad.0cfxpnw3c. The Bayesian trees are available from the Dryad Digital Repository at https://doi.org/10.5061/dryad.rv15dv48m. The haplotype sequences of our study involved are deposited in GenBank with accession numbers MZ130327‐MZ130377.

## References

[ece38517-bib-0001] Arnold, M. L. , Ballerini, E. S. , & Brothers, A. N. (2012). Hybrid fitness, adaptation and evolutionary diversification: Lessons learned from *Louisiana irises* . Heredity, 108, 159–166. 10.1038/hdy.2011.65 21792222 PMC3282389

[ece38517-bib-0002] Arnold, M. L. , & Martin, N. H. (2009). Adaptation by introgression. Journal of Biology, 8, 82. 10.1186/jbiol176 19833002 PMC2776903

[ece38517-bib-0003] Barkwoth, M. E. , & Bothmer, R. V. (2009). Scientific names in the Triticeae. Genetics and Genomics of the Triticeae, 7, 3–30. 10.1007/978-0-387-77489-3_1

[ece38517-bib-0004] Barton, N. H. (2001). The role of hybridization in evolution. Molecular Ecology, 10(3), 551–568. 10.1046/j.1365-294x.2001.01216.x 11298968

[ece38517-bib-0005] Brysting, A. K. , Holst‐Jensen, A. , & Leitch, I. (2000). Genomic origin and organization of the hybrid *Poa jemtlandica* (Poaceae) verified by genomic in situ hybridization and chloroplast DNA sequences. Annals of Botany, 85(4), 439–445. 10.1006/anbo.1999.1088

[ece38517-bib-0006] Buerkle, C. A. , Morris, R. J. , Asmussen, M. A. , & Rieseberg, L. H. (2000). The likelihood of homoploid hybrid speciation. Heredity, 84(4), 441–451. 10.1046/j.1365-2540.2000.00680.x 10849068

[ece38517-bib-0007] Dewey, D. R. (1984). The genomic system of classification as a guide to intergeneric hybridization with the perennial Triticeae. In J. P. Gustafson (Ed.), Gene manipulation in plant improvement (pp. 209–279). Columbia University Press. 10.1007/978-1-4613-2429-4_9

[ece38517-bib-0008] Felsenstein, J. (1985). Confidence limits on phylogenies: An approach using the bootstrap. Evolution, 39(4), 783–791. 10.1111/j.15585646.1985.tb00420.x 28561359

[ece38517-bib-0009] Gaeta, R. T. , & Pires, J. C. (2010). Homologous recombination in allopolyploids: the polyploid ratchet. New Phytologist, 186, 18–28. 10.1111/j.1469-8137.2009.03089.x 20002315

[ece38517-bib-0010] Gill, B. S. , Friebe, B. , & Endo, T. R. (1991). Standard karyotype and nomenclature system for description of chromosome bands and structural aberrations in wheat (*Triticum aestivum*). Genome, 34, 830–839. 10.1139/G91-128

[ece38517-bib-0011] Gross, B. L. , & Rieseberg, L. H. (2005). The ecological genetics of homoploid hybrid speciation. Journal of Heredity, 96(3), 241–252. 10.1093/jhered/esi026 PMC2517139 15618301

[ece38517-bib-0012] Guindon, S. , Delsuc, F. , Dufayard, J. F. , & Gascuel, O. (2009). Estimating maximum likelihood phylogenies with PhyML. Methods in Molecular Biology, 537, 113–137. 10.1007/978-1-59745-251-9_6 19378142

[ece38517-bib-0013] Han, F. , Liu, B. , Fedak, G. , & Liu, Z. (2004). Genomic constitution and variation in five partial amphiploids of wheat – *Thinopyrum intermedium* as revealed by GISH, multicolor GISH and seed storage protein analysis. Theoretical and Applied Genetics, 109(5), 1070–1076. 10.1007/s00122-004-1720-y 15197444

[ece38517-bib-0014] Katoh, K. , & Standley, D. M. (2013). MAFFT multiple sequence alignment software version 7: improvements in performance and usability. Molecular Biology and Evolution, 30(4), 772–780. 10.1093/molbev/mst010 23329690 PMC3603318

[ece38517-bib-0015] Keng, Y. (1959). Flora Illustrata Plantarum Primarum Sinicarum (Gramineae). Science Press.

[ece38517-bib-0016] Kunte, K. , Shea, C. , Aardema, M. L. , Scriber, J. M. , Juenger, T. E. , Gilbert, L. E. , & Kronforst, M. R. (2011). Sex chromosome mosaicism and hybrid speciation among tiger swallowtail butterflies. PLoS Genetics, 7, e1002274. 10.1371/journal.pgen.1002274 21931567 PMC3169544

[ece38517-bib-0017] Lei, Y.‐X. , Liu, J. , Fan, X. , Sha, L.‐N. , Wang, Y. I. , Kang, H.‐Y. , Zhou, Y.‐H. , & Zhang, H.‐Q. (2018). Phylogeny and molecular evolution of the *DMC*1 gene in the polyploid genus *Roegneria* and its affinitive genera (Poaceae: Triticeae). Botanical Journal of the Linnean Society, 186(1), 129–142. 10.1093/botlinnean/box081

[ece38517-bib-0018] Mallet, J. (2005). Hybridization as an invasion of the genome. Trends in Ecology and Evolution, 20(5), 229–237. 10.1016/j.tree.2005.02.010 16701374

[ece38517-bib-0019] Mallet, J. (2007). Hybrid speciation. Nature, 446(7133), 279–283. 10.1038/nature05706 17361174

[ece38517-bib-0020] Mao, J. , Ma, Y. , & Zhou, R. C. (2017). Approaches used to detect and test hybridization: combining phylogenetic and population genetic analyses. Biodiversity Science, 25(6), 577–599. 10.17520/biods.2017097

[ece38517-bib-0021] Martis, M. M. , Zhou, R. , Haseneyer, G. , Schmutzer, T. , Vrána, J. , Kubaláková, M. , König, S. , Kugler, K. G. , Scholz, U. , Hackauf, B. , Korzun, V. , Schön, C.‐C. , Doležel, J. , Bauer, E. , Mayer, K. F. X. , & Stein, N. (2013). Reticulate evolution of the rye genome. The Plant Cell, 25(10), 3685–3698. 10.1105/tpc.113.114553 24104565 PMC3877785

[ece38517-bib-0022] Murray, H. G. , & Thompson, W. F. (1980). Rapid isolation of high molecular weight plant DNA. Nucleic Acids Research, 8(19), 4321–4326. 10.1093/nar/8.19.4321 7433111 PMC324241

[ece38517-bib-0023] Nolte, A. W. , & Tautz, D. (2010). Understanding the onset of hybrid speciation. Trends in Genetics, 26(2), 54–58. 10.1016/j.tig.2009.12.001 20044166

[ece38517-bib-0024] Paštová, L. , Belyayev, A. , & Mahelka, V. (2019). Molecular cytogenetic characterisation of *Elytrigia* ×*mucronata*, a natural hybrid of *E. intermedia* and *E. repens* (Triticeae, Poaceae). BMC Plant Biology, 19(1): 230. 10.1186/s12870-019-1806-y 31151385 PMC6544950

[ece38517-bib-0025] Petersen, G. , & Seberg, O. (2002). Molecular evolution and phylogenetic application *DMC*1. Molecular Phylogenetics and Evolution, 22(1), 43–50. 10.1006/mpev.2001.1011 11796028

[ece38517-bib-0026] Posada, D. , & Crandall, K. A. (1998). MODELTEST: Testing the model of DNA substitution. Bioinformatics, 14(9), 817–818. 10.1093/bioinformatics/14.9.817 9918953

[ece38517-bib-0027] Rieseberg, L. H. (1995). The role of hybridization in evolution: Old wine in new skins. American Journal of Botany, 82(7), 944–953. 10.1002/J.1537-2197.1995.TB15711.X

[ece38517-bib-0028] Rieseberg, L. (1997). Hybrid origin of plant species. Annual Review of Ecology and Systematics, 28, 359–389. 10.1146/annurev.ecolsys.28.1.359

[ece38517-bib-0029] Rieseberg, L. H. , Ellstrand, N. C. , & Arnold, M. (1993). What can molecular and morphological markers tell us about plant hybridization? Critical Reviews in Plant Science, 12(3), 213–241. 10.1080/07352689309701902

[ece38517-bib-0030] Rieseberg, L. H. , & Willis, J. H. (2007). Plant speciation. Science, 317(5840), 910–914. 10.1126/science.1137729 17702935 PMC2442920

[ece38517-bib-0031] Ronquist, F. , & Huelsenbeck, J. P. (2003). MRBAYES 3: Bayesian phylogenetic inference under mixed model. Bioinformatics, 19(12), 1572–1574. 10.1093/bioinformatics/btg180 12912839

[ece38517-bib-0032] Sha, L. , Fan, X. , Yang, R. , Kang, H. , Ding, C. , Zhang, L. I. , Zheng, Y. , & Zhou, Y. (2010). Phylogenetic relationships between *Hystrix* and its closely related genera (Triticeae; Poaceae) based on nuclear *Acc*1, *DMC*1 and chloroplast *trnL*‐*F* sequences. Molecular Phylogenetics and Evolution, 54(2), 327–335. 10.1016/j.ympev.2009.05.005 19435606

[ece38517-bib-0033] Shaw, J. , Lickey, E. B. , Beck, J. T. , Farmer, S. B. , Liu, W. , Miller, J. , Siripun, K. C. , Winder, C. T. , Schilling, E. E. , & Small, R. L. (2005). The tortoise and the hare II: Relative utility of 21 noncoding chloroplast DNA sequences for phylogenetic analysis. American Journal of Botany, 92(1), 142–166. 10.3732/ajb.92.1.142 21652394

[ece38517-bib-0034] Smith, J. F. , Funke, M. M. , & Woo, V. L. (2006). A duplication of *gcyc* predates divergence within tribe *Coronanthereae* (Gesneriaceae): Phylogenetic analysis and evolution. Plant Systematics and Evolution, 261(1–4), 245–256. 10.1007/s00606-006-0445-6

[ece38517-bib-0035] Soltis, D. E. , Clayton, J. V. , & Soltis, P. S. (2014). The polyploidy revolution then…and now: Stebbins revisited. American Journal of Botany, 101(7), 1057–1078. 10.3732/ajb.1400178 25049267

[ece38517-bib-0036] Soltis, P. S. , Doyle, J. J. , & Soltis, D. E. (1992). Molecular data and polyploid evolution in plants. In P. S. Soltis , D. E. Soltis , & J. J. Doyle (Eds.), Molecular systematics of plant (pp. 177–201). Chapman & Hall. 10.1007/978-1-4615-3276-7_8

[ece38517-bib-0037] Soltis, P. S. , & Soltis, D. E. (2009). The role of hybridization in plant speciation. Annual Review of Plant Biology, 60, 561–588. 10.1146/annurev.arplant.043008.092039 19575590

[ece38517-bib-0038] Tang, C. , Qi, J. , Chen, N. , Sha, L.‐N. , Wang, Y. I. , Zeng, J. , Kang, H.‐Y. , Zhang, H.‐Q. , Zhou, Y.‐H. , & Fan, X. (2017). Genome origin and phylogenetic relationships of *Elymus villosus* (Triticeae: Poaceae) based on single‐copy nuclear *Acc*1, *Pgk*1, *DMC*1 and chloroplast *trnL*‐*F* sequences. Biochemical Systematics and Ecology, 70, 168–176. 10.1016/j.bse.2016.11.011

[ece38517-bib-0039] von Bothmer, R. , & Salomon, B. (1994). Triticeae: A tribe for food, feed and fun. Herbarium Publications, 24. https://digitalcommons.usu.edu/herbarium_pubs/24

[ece38517-bib-0040] Wang, L. , Shi, Q. , Su, H. , Wang, Y. , Sha, L. , Fan, X. , & Zhou, Y. (2017). St2‐80: A new FISH marker for **St** genome and genome analysis in Triticeae. Genome, 60(7), 553–563. 10.1139/gen-2016-0228 28314114

[ece38517-bib-0041] White, O. W. , Reyes‐Betancort, A. , Chapman, M. A. , & Carine, M. A. (2018). Independent Homoploid Hybrid Speciation events in the Macaronesian endemic genus *Argyranthemum* . Molecular Ecology, 27(23): 4856–4874. 10.3732/ajb.92.1.142 30281862

[ece38517-bib-0042] Whitney, K. D. , Randell, R. A. , & Rieseberg, L. H. (2010). Adaptive introgression of abiotic tolerance traits in the sunflower *Helianthus annuus* . New Phytologist, 187(1), 230–239. 10.1111/j.1469-8137.2010.03234.x 20345635

[ece38517-bib-0043] Yan, C. , Hu, Q. , Sun, G. , & McIntyre, C. L. (2014). Nuclear and chloroplast DNA phylogeny reveals complex evolutionary history of *Elymus pendulinus* . Genome, 57(2), 97–109. 10.1139/gen-2014-0002 24702067

[ece38517-bib-0044] Yang, J. L. , Baum, B. R. , & Yen, C. (2008). A revision of the genus *Roegneria* C. Koch (Poaceae: Triticeae). Journal of Sichuan Agricultural University, 26, 311–381.

[ece38517-bib-0045] Zhang, H. , Fan, X. , Wang, Y. , & Zhou, Y. (2008). Cytogenetic studies of intergeneric hybrids between *Roegneria ciliaris* and *Leymus multicaulis* (Poaceae: Triticeae). Acta Prataculturae Sinica, 17, 162–165.

[ece38517-bib-0046] Zhang, H. , & Zhou, Y. (2006). Meiotic pairing behaviour reveals differences in genomic constitution between *Hystrix patula* and other species of genus *Hystrix* Moench (Poaceae, Triticeae). Plant Systematics and Evolution, 258, 129–136. 10.1007/s00606-005-0394-5

[ece38517-bib-0047] Zhou, Y. , Yen, C. , & Yang, J. (1995). A study on the intergeneric hybrid of *R. kamoji* × *Hordeum vulgare* . Journal of Sichuan Agricultural University, 13, 144–149.

[ece38517-bib-0048] Zhou, Y. , Yen, C. , Yang, J. , & Zheng, Y. (1999). Biosystematic study of *Roegneria tenuispica*, *R. ciliaris* and *R. pendulina* (Poaceae: Triticeae). Plant Systematics and Evolution, 217, 215–220. 10.1007/BF00984367

