# Supplementary material for: Morphological, cytological, and molecular evidences for natural hybridization between Roegneria stricta and Roegneria turczaninovii (Triticeae: Poaceae)
Source: Ecol Evol. 2022 Jan 12;12(1):e8517. doi: 10.1002/ece3.8517 (PMC8809439; doi:10.1002/ece3.8517)
Supplement: Supplementary file 1 — Table S1‐S2 [file ECE3-12-e8517-s001.docx]

**supplementary material**

**TABLE S1** The primers used in this study

| Gene | Name of primers | Sequence of primer (5´- 3´) | Profiles |
| --- | --- | --- | --- |
| *DMC*1 | T*DMC*1e10F | TGCCAATTGCTGAGAGATTTG | 1 cycle: 4 min 94 ℃; 35 cycles: 1 min 94 ℃, 1 min 52 ℃, 2 min 40 s 72 ℃; 1 cycle: 10 min 72 ℃ |
|  | T*DMC*1e15R | AGCCACCTGTTGTAATCTGG |  |
| *rps*16 | *rps*16F | AAACGATGTGGTAGAAAGCAAC | 1 cycle: 3 min 95 ℃; 35 cyclies: 40 s 94 ℃, 40s 55 ℃, 1 min 40 s 72 ℃; 1 cycle: 10 min 72 ℃ |
|  | *rps*16R | ACATCAATTGCAACGATTCGATA |  |

**TABLE S2** Meiotic associations at metaphase I in PMCs of the parental species and hybrid

| Species and hybrids | No. of cells observed | Chromosome pairing | | | | | Chiasmata cell | C value |
| --- | --- | --- | --- | --- | --- | --- | --- | --- |
|  |  | I | Ring II | Rod II | Total II | IV |  |  |
| *R. stricta* | 463 | – | 13.55 | 0.44 | 13.99 | 0.01 | 27.60 | 0.99 |
| *R. turczaninovii* | 198 | – | 13.53 | 0.43 | 13.96 | 0.02 | 27.57 | 0.98 |
| RH1-1 | 24 | 1.60 | 11.45 | 1.75 | 13.20 | – | 24.65 | 0.81 |
| RH1-2 | 28 | 0.90 | 12.05 | 1.60 | 13.65 | – | 25.70 | 0.92 |
| RH1-3 | 21 | 0.60 | 12.15 | 1.55 | 13.70 | – | 25.85 | 0.92 |
| RH1-4 | 35 | 1.90 | 11.10 | 1.95 | 13.05 | – | 24.15 | 0.86 |
| RH1-5 | 23 | 0.93 | 10.98 | 2.53 | 13.51 | – | 24.49 | 0.87 |
| RH1-6 | 24 | 0.50 | 12.15 | 1.65 | 13.80 | – | 25.95 | 0.93 |
| RH1-7 | 20 | 1.10 | 10.85 | 2.60 | 13.45 | – | 24.30 | 0.87 |
| RH1-8 | 34 | 0.53 | 11.44 | 2.29 | 13.73 | – | 25.17 | 0.90 |
| RH1-9 | 20 | 1.30 | 11.75 | 1.60 | 13.35 | – | 25.10 | 0.90 |
| RH1-10 | 20 | 1.00 | 10.70 | 2.80 | 13.50 | – | 24.20 | 0.86 |
| RH1-11 | 25 | 0.50 | 12.15 | 1.60 | 13.75 | – | 25.90 | 0.93 |
| RH1-12 | 21 | 0.90 | 11.75 | 1.80 | 13.55 | – | 25.30 | 0.90 |
| RH1-13 | 35 | 0.51 | 11.97 | 1.77 | 13.74 | – | 25.71 | 0.92 |
| RH1-14 | 20 | 2.00 | 11.50 | 1.50 | 13.00 | – | 24.50 | 0.88 |
| RH1-15 | 20 | 1.20 | 11.15 | 2.25 | 13.40 | – | 24.55 | 0.88 |
| RH1-16 | 20 | 0.80 | 12.00 | 1.60 | 13.60 | – | 25.60 | 0.91 |
| RH1-17 | 32 | 0.42 | 12.12 | 1.67 | 13.79 | – | 25.91 | 0.93 |
| RH2-1 | 20 | 0.60 | 11.50 | 2.20 | 13.70 | – | 25.20 | 0.90 |
| RH2-2 | 20 | 0.50 | 12.70 | 1.05 | 13.75 | – | 26.45 | 0.94 |
| RH2-3 | 20 | 1.10 | 11.05 | 2.40 | 13.45 | – | 24.50 | 0.88 |
| RH2-4 | 20 | 0.50 | 11.65 | 2.10 | 13.75 | – | 25.40 | 0.91 |
| RH2-5 | 23 | 1.30 | 11.10 | 2.25 | 13.35 | – | 24.45 | 0.87 |
| RH2-6 | 20 | 2.15 | 11.30 | 2.15 | 13.45 | – | 24.75 | 0.88 |
| RH2-7 | 31 | 1.70 | 11.11 | 2.05 | 12.97 | – | 24.27 | 0.87 |
| RH2-8 | 20 | 1.10 | 11.50 | 1.95 | 13.45 | – | 24.95 | 0.89 |
| RH2-9 | 22 | 1.30 | 11.40 | 1.95 | 13.35 | – | 24.75 | 0.88 |
| RH2-10 | 20 | 0.70 | 10.60 | 2.35 | 12.95 | – | 23.55 | 0.84 |
| RH2-11 | 24 | 3.45 | 11.25 | 2.05 | 13.30 | – | 24.55 | 0.87 |
| RH2-12 | 20 | 0.50 | 12.25 | 1.45 | 13.70 | – | 25.95 | 0.93 |
| RH2-13 | 20 | 0.90 | 11.20 | 2.35 | 13.55 | – | 24.75 | 0.88 |
| RH2-14 | 20 | 0.85 | 11.20 | 2.45 | 13.65 | – | 24.85 | 0.89 |
| RH2-15 | 20 | 2.10 | 10.60 | 2.35 | 12.95 | – | 23.55 | 0.84 |
| RH2-16 | 20 | 0.50 | 10.95 | 2.80 | 13.75 | – | 24.70 | 0.88 |
| RH2-17 | 20 | 0.95 | 10.70 | 2.85 | 13.55 | – | 24.25 | 0.87 |
| RH2-18 | 20 | 0.70 | 11.60 | 2.05 | 13.65 | – | 25.25 | 0.90 |
| RH2-19 | 20 | 1.20 | 12.20 | 1.20 | 13.40 | – | 25.60 | 0.91 |
| RH2-20 | 20 | 0.10 | 11.50 | 2.45 | 13.95 | – | 25.45 | 0.91 |
| RH2-21 | 19 | 0.42 | 11.32 | 2.21 | 13.53 | – | 24.85 | 0.89 |
| RH2-22 | 47 | 0.46 | 12.66 | 1.07 | 13.73 | – | 26.39 | 0.94 |
| RH2-23 | 20 | 0.20 | 11.75 | 2.15 | 13.90 | – | 25.65 | 0.92 |
| RH2-24 | 20 | 0.40 | 11.45 | 2.35 | 13.80 | – | 25.25 | 0.90 |
| RH2-25 | 27 | 0.20 | 12.00 | 1.90 | 13.90 | – | 25.90 | 0.93 |
| RH2-26 | 35 | 0.60 | 11.85 | 1.85 | 13.70 | – | 25.55 | 0.91 |
| RH2-27 | 21 | 0.50 | 11.95 | 1.80 | 13.75 | – | 25.70 | 0.92 |
| RH2-28 | 24 | 1.00 | 11.95 | 1.55 | 13.50 | – | 25.45 | 0.91 |
| RH2-29 | 22 | 0.80 | 11.65 | 2.05 | 13.70 | – | 25.35 | 0.91 |
| RH2-30 | 26 | 0.40 | 11.80 | 2.00 | 13.80 | – | 25.60 | 0.91 |
| RH2-31 | 31 | – | 11.55 | 2.45 | 14.00 | – | 25.55 | 0.91 |
| RH2-32 | 38 | 0.68 | 11.82 | 1.87 | 13.69 | – | 25.51 | 0.91 |
| RH2-33 | 37 | 0.70 | 11.81 | 1.81 | 13.62 | – | 25.43 | 0.92 |
| RH2-34 | 24 | 0.80 | 11.35 | 2.25 | 13.60 | – | 24.95 | 0.89 |
| RH2-35 | 24 | 0.50 | 11.69 | 1.98 | 13.67 | – | 25.36 | 0.91 |
| RH2-36 | 40 | 1.20 | 11.65 | 1.75 | 13.40 | – | 25.05 | 0.89 |
| RH2-37 | 23 | 0.80 | 12.65 | 0.85 | 13.50 | – | 26.15 | 0.93 |
| RH2-38 | 19 | 1.48 | 11.32 | 1.92 | 13.24 | – | 24.56 | 0.88 |
| RH2-39 | 48 | 0.58 | 11.81 | 2.10 | 13.91 | – | 25.72 | 0.92 |
| RH2-40 | 39 | 0.10 | 12.21 | 1.72 | 13.93 | – | 26.14 | 0.93 |
